# Oral hygiene practices in nurseries (0-3 years) in the cities of Pavia and Vigevano

**DOI:** 10.3389/froh.2022.991741

**Published:** 2023-01-18

**Authors:** Marzia Segù, Laura Baroni, Federica Bertuzzi, Camilla Preda

**Affiliations:** ^1^ Department of Medicine and Surgery, University of Parma, Parma, Italy; ^2^ Department of Clinical-Surgical, Diagnostic and Pediatric Sciences, Section of Dentistry, University of Pavia, Pavia, Italy

**Keywords:** pediatric oral hygiene, prevention, nurseries, dental hygienist, diet

## Abstract

**Aim:**

This study was conducted to evaluate attention to and knowledge of oral care in children aged 0–3 years, on the basis of data collected from early years educators working with this age group. Information was also collected about the oral hygiene practices adopted in nurseries and the types of food provided, with the aim of increasing knowledge and raising awareness of this topic.

**Materials and methods:**

A questionnaire was created using the “Google Forms” program and sent to all the 47 nurseries in the city of Pavia and Vigevano.

**Results:**

Twenty establishments agreed to take part in the research. Assessment of knowledge and of awareness of oral care among early years educators caring for children in the age range 0–3 years revealed variability and some confusion. The results showed a general lack of attention to oral care in the period before the milk teeth appear, as well as limited use of gauze swabs. There was generally some use of educational play focusing on this issue, albeit not daily across all the nurseries participating in the survey. Nurseries rarely had the support of a professional dental hygienist to raise awareness among early years educators and parents.

**Conclusions:**

The findings obtained through this study indicate that, in the area surveyed, there is a lack of widespread knowledge and awareness of oral prevention in young children, and of the oral hygiene maintenance techniques to use in this age group. This suggests a need to develop preventive protocols to improve knowledge and awareness of children's oral health among the adults who care for them.

## Introduction

At birth the oral cavity is sterile, but from the first moments of life it is colonized by bacteria that become commensal microorganism.

Oral hygiene is crucial to maintaining a healthy mouth at any age and entails cleaning both the hard structures of the tooth and the soft tissues (mucosa and gingiva) to help eliminate food residues and bacteria that have formed. The aim of oral hygiene is to maintain a correct balance between oral bacteria and the host organism.

From birth, oral hygiene is therefore a crucial factor in preventing diseases such as caries and periodontal disease. It is sometimes neglected in infants prior to the appearance of the first teeth, but this is a mistake as there is always some sugar in the milk, of whatever type, administered to infants.

The cleaning technique used should be the one most appropriate for the child's age: for infants aged from 0 to around 7–8 months, a gauze pad soaked in physiological saline solution or water can be used to gently rub the gums, cleaning away bacterial plaque, traces of milk and, once the child is being weaned, also food residue; thereafter, as the first teeth come in, specially designed silicone finger toothbrushes can be used instead; after the age of 24 months, it is recommended to use a toothbrush.

Another key aspect in prevention is diet: foods rich in highly acidic sugars, in addition to being incorrect from a nutritional point of view, encourage tooth decay, especially of the first teeth as the primary enamel is less mineralised than that of permanent teeth.

Studying infants and children aged 0–3 years is particularly difficult due to lack of collaboration. Parents and early life practitioners caring for children need to receive appropriate information from professionals, so that they are properly instructed and aware of the dietary aspects of oral care and of the oral hygiene practices that should be implemented.

The aim of this study was to evaluate, by means of a questionnaire, early years educators' knowledge on the topic of oral hygiene in children of the age they work with. Information on the oral hygiene practices and routines adopted in the nurseries was collected, as well as on the provision of appropriate foods and educational activities designed to encourage oral hygiene. When caregivers establish good habits at an early age, children are more likely to acquire them later on. Through awareness-raising activities, parents and educators can become more capable and better informed.

## Materials and methods

Our research work involved the development of an original questionnaire, including both open and closed questions, aimed at nursery owners. The aim was to evaluate the level of attention to oral hygiene and any oral hygiene-related activities carried out within these educational facilities. The questionnaire was also designed to evaluate interest in a possible collaboration with an oral care specialist.

The questionnaire was created using Google Forms and sent anonymously to all the 47 nurseries in the city of Pavia and in the city of Vigevano.

## Results

The questionnaire was sent to all nurseries in the city of Pavia and Vigevano. Only twenty agreed to take part in the study. The results of the study are summarized in the following figures (Figures 1–14).

**Question 1 Figure 1:**
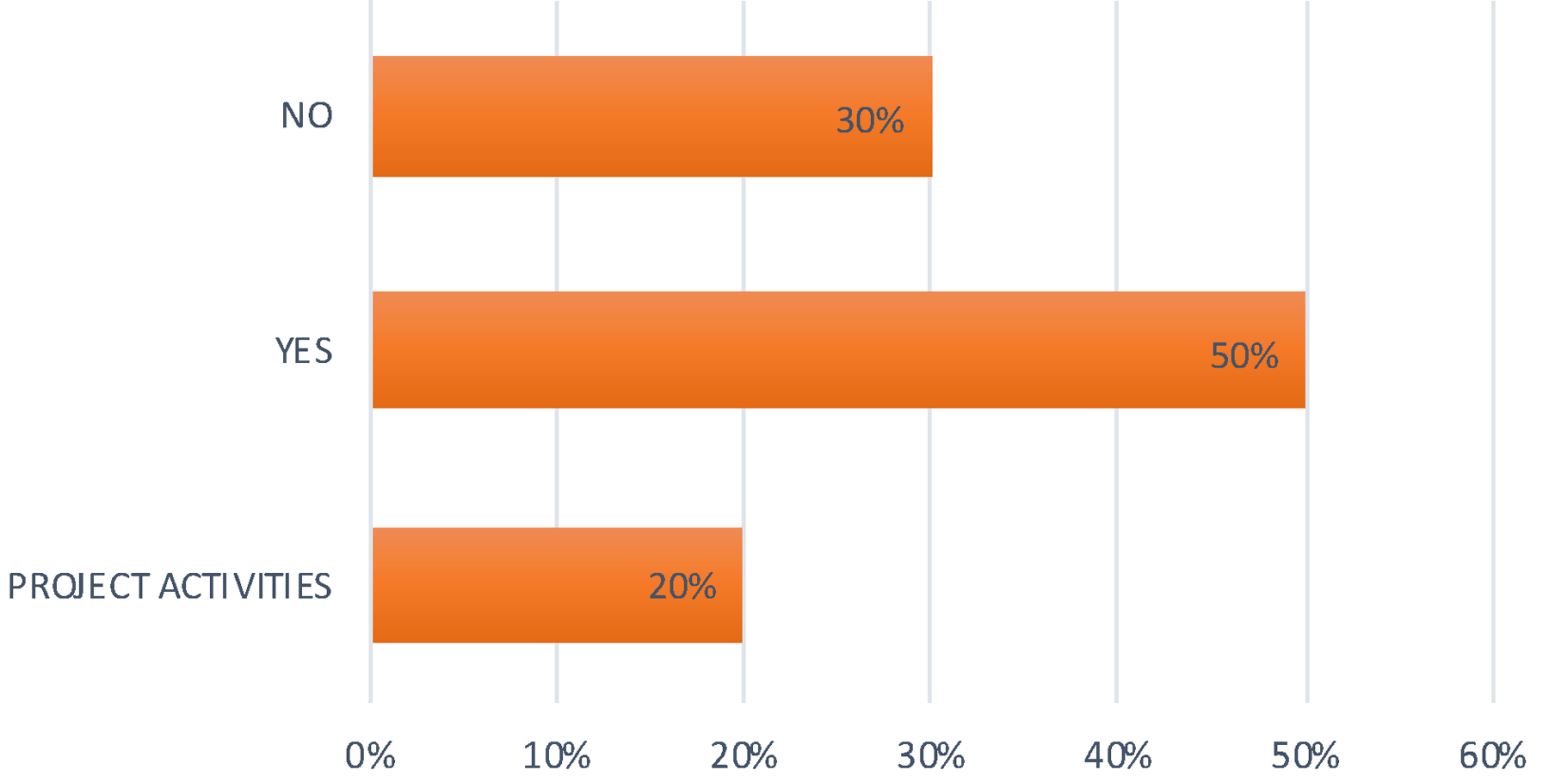
*Is oral hygiene practised at your nursery?* 50% of the nurseries replied yes; 30% replied no; 20% have carried out oral hygiene activities on a project basis or have discntinued them due to Covid.

**Question 2 Figure 2:**
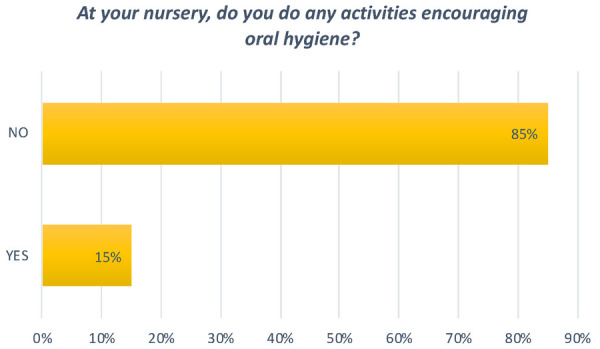
*At your nursery, do you do any activities encouraging oral hygiene?* 15% answered no. The other 85% answered yes, with books and games being the most popular means of communication used.

**Question 3 Figure 3:**
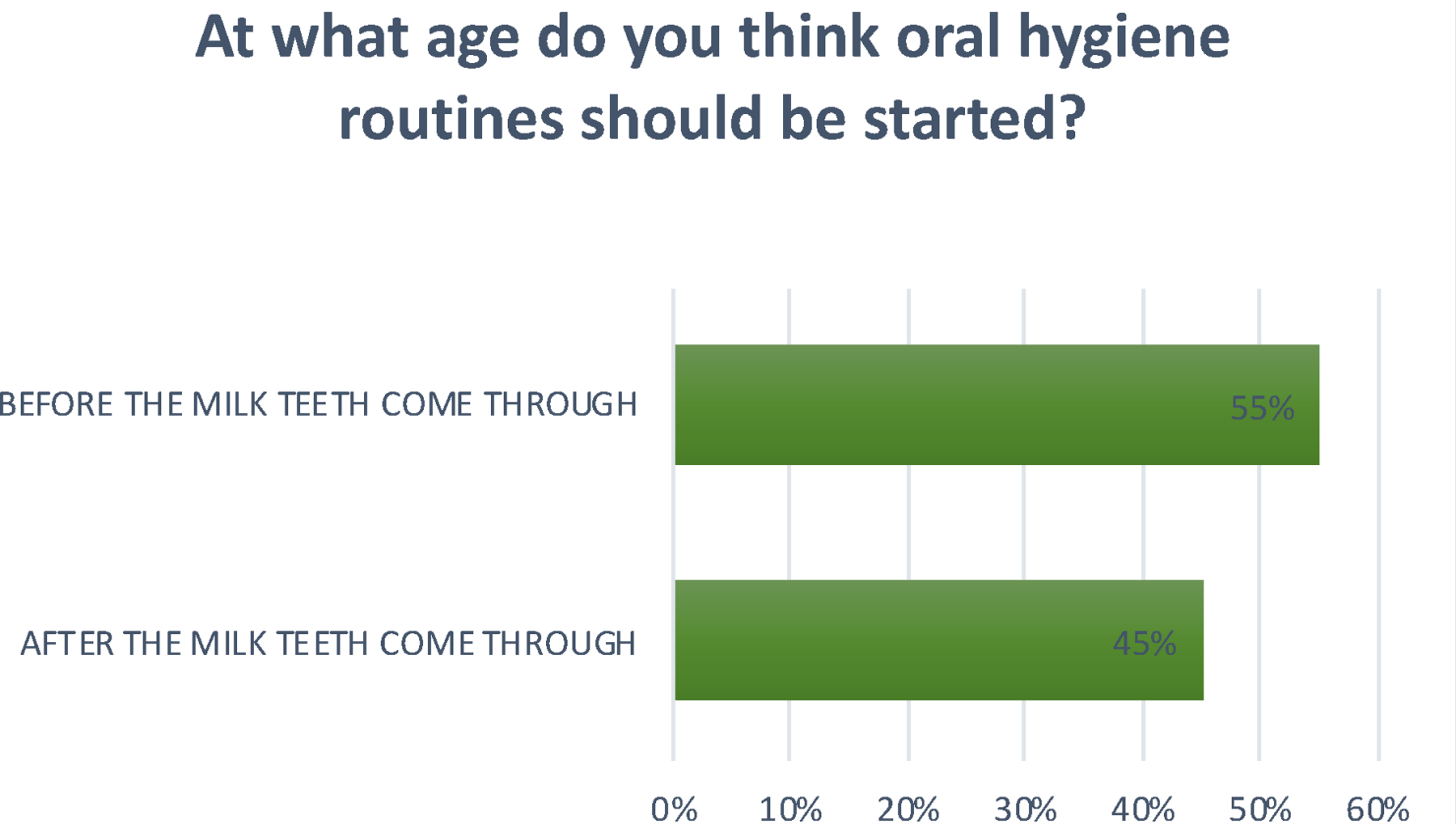
*At what age do you think oral hygiene routines should be started?* 55% replied before the milk teeth come through; 45% replied after the milk teeth come through.

**Question 4 Figure 4:**
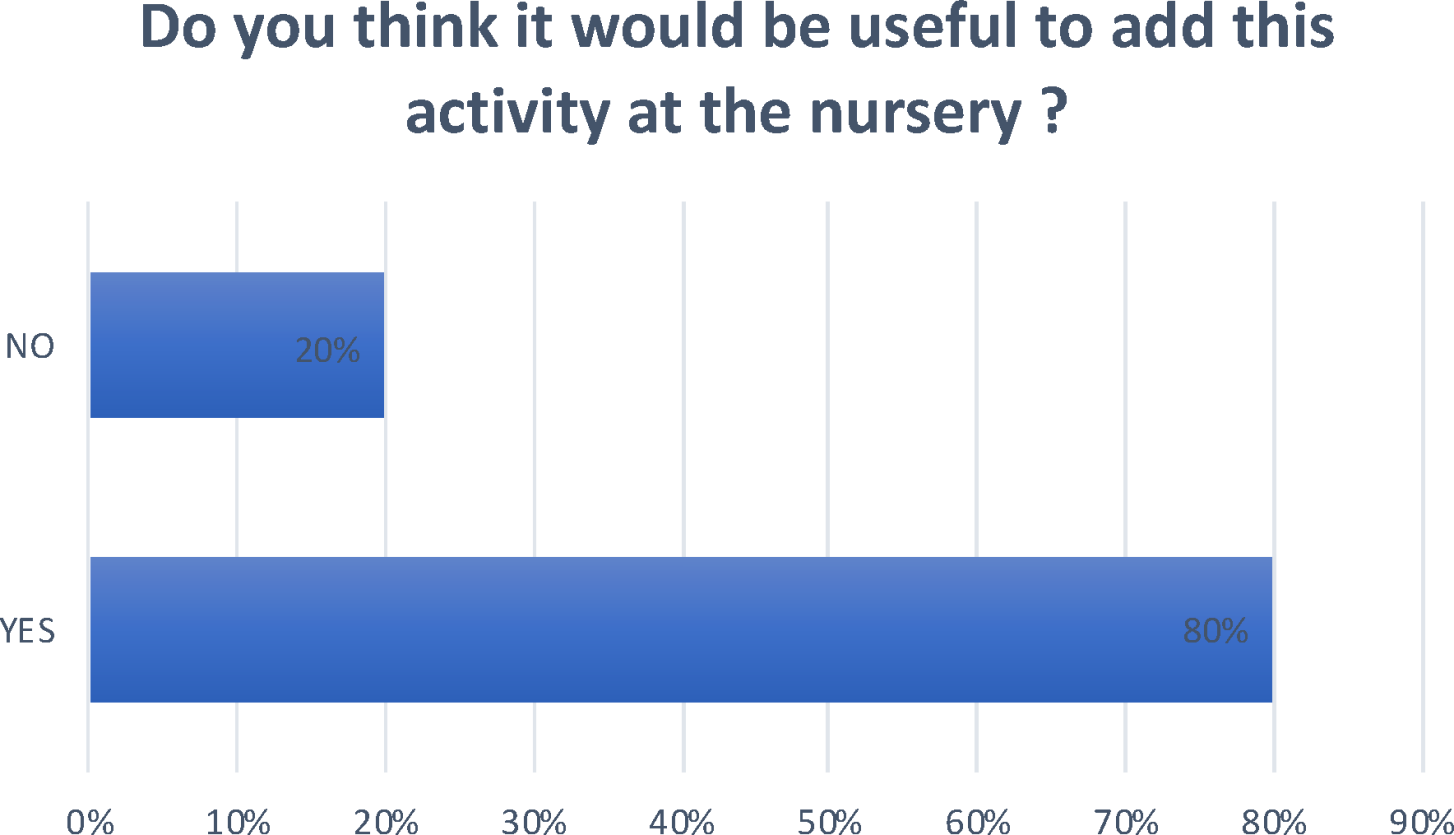
*Do you think it would be useful to add this activity at the nursery?* 80% answered yes; 20% answered no.

**Question 5 Figure 5:**
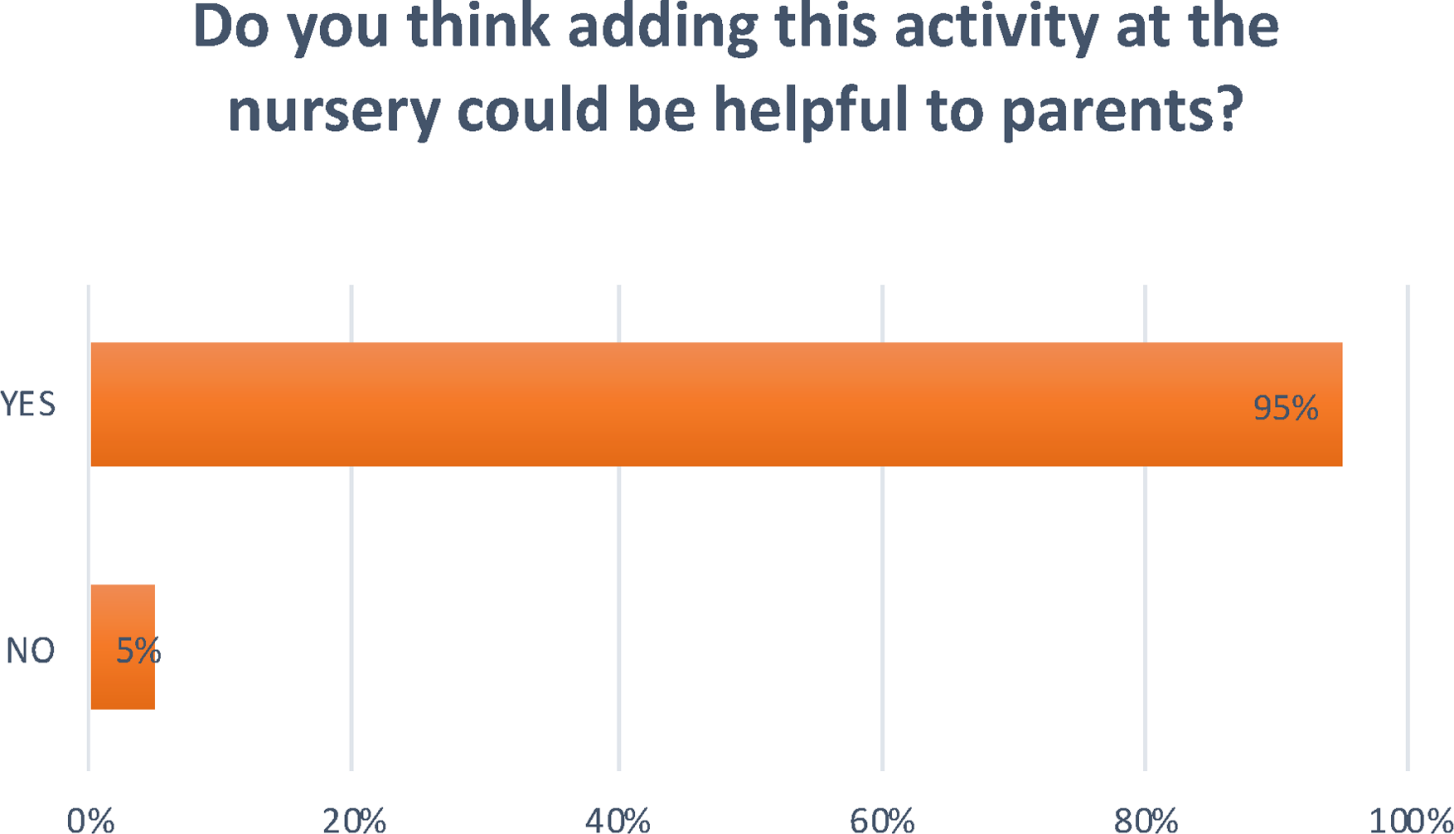
*Do you think adding this activity at the nursery could be helpful to parents?* 95% answered yes; 5% answered no.

**Question 6 Figure 6:**
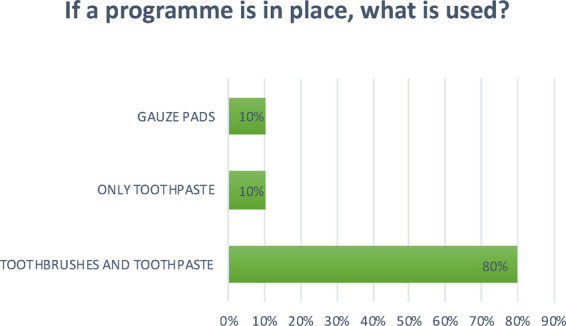
*If a program is in place, what is used?* 80% of the nurseries surveyed replied that they use toothbrushes and toothpaste; 10% use only toothpaste; 10% use gauze pads.

**Question 7 Figure 7:**
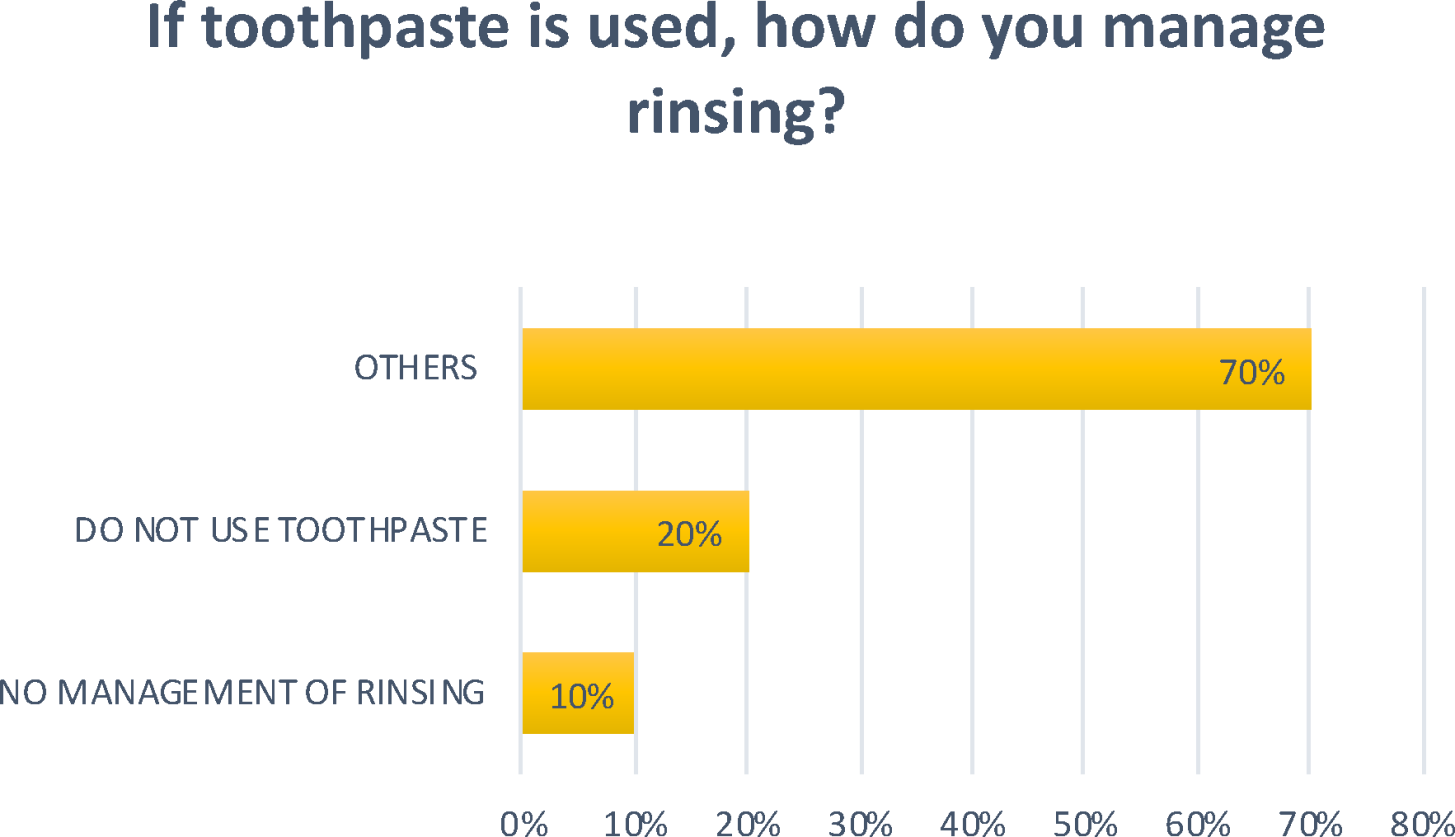
*If toothpaste is used, how do you manage rinsing?* 10% replied that there is no management of rinsing; 20% replied that they do not use toothpaste; The remaining percentage includes nurseries that use non-fluoride toothpaste and ones that try to teach children to spit.

**Question 8 Figure 8:**
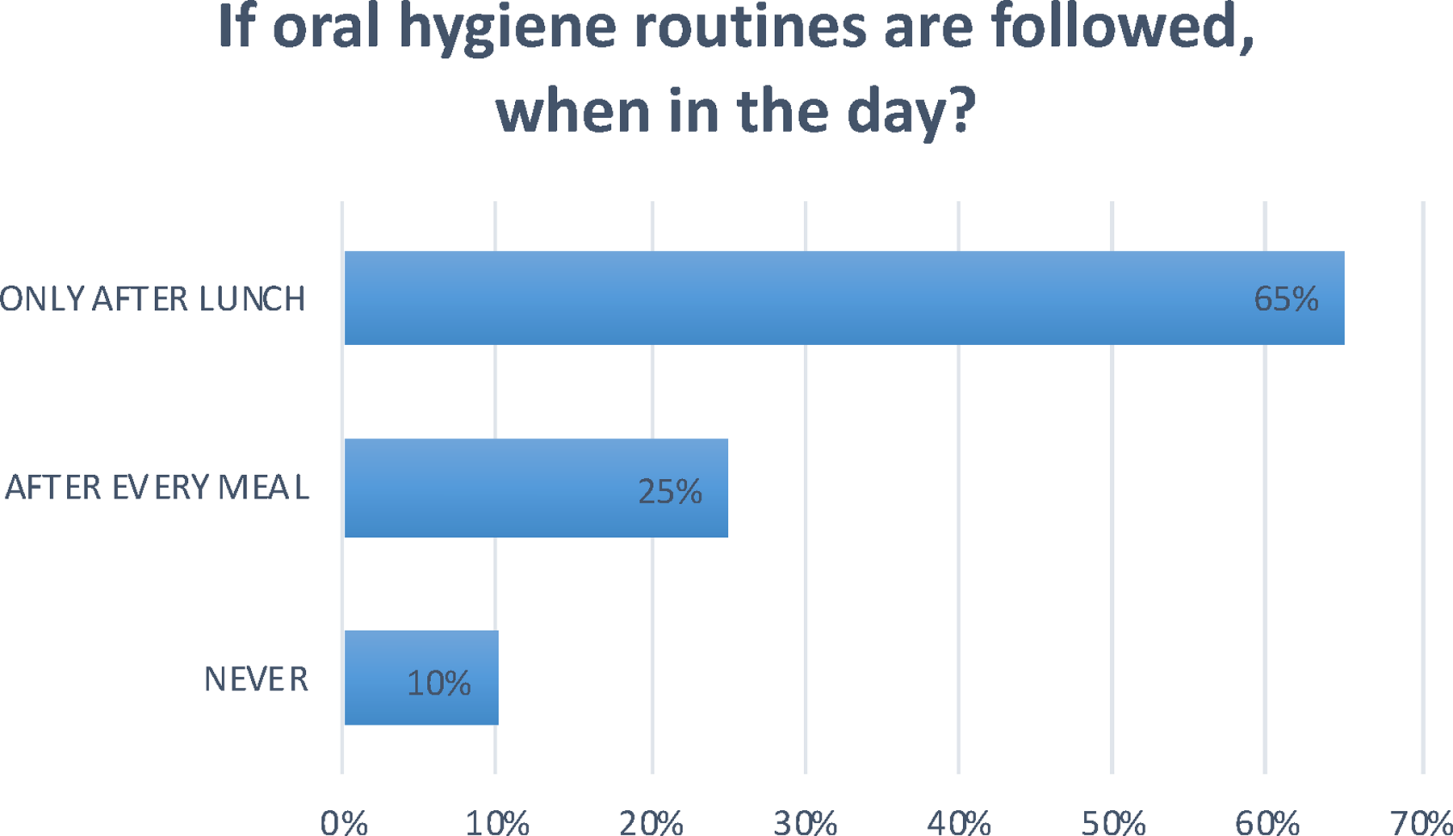
*If oral hygiene routines are followed, when in the day?* In 25% of the nurseries oral hygiene routines are followed after every meal; In 65% of the nurseries only after lunch; In 10% no oral hygiene routines are followed.

**Question 9 Figure 9:**
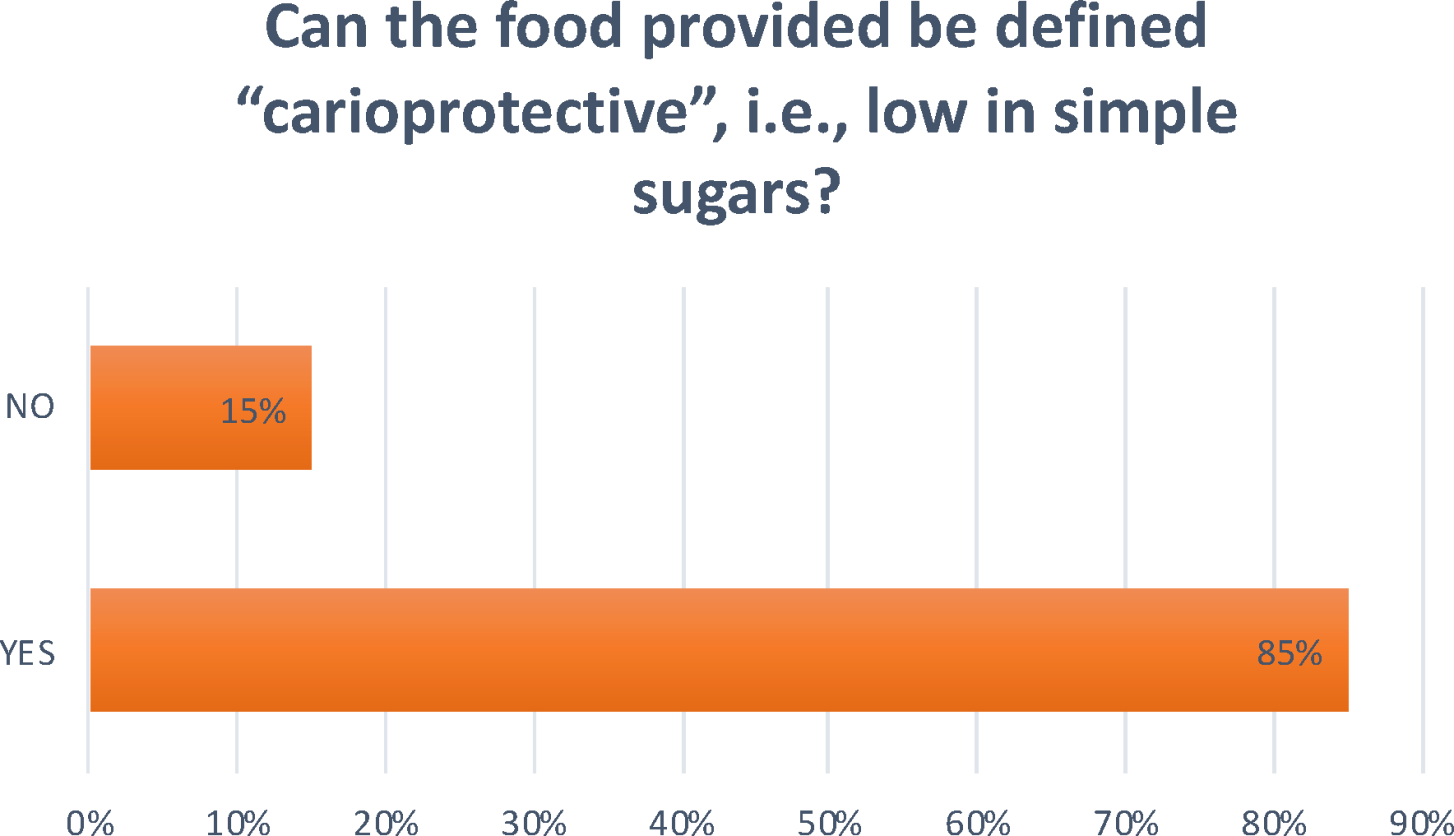
*Can the food provided be defined “carioprotective”, i.e., low in simple sugars?* 85% replied yes; 15% replied no.

**Question 10 Figure 10:**
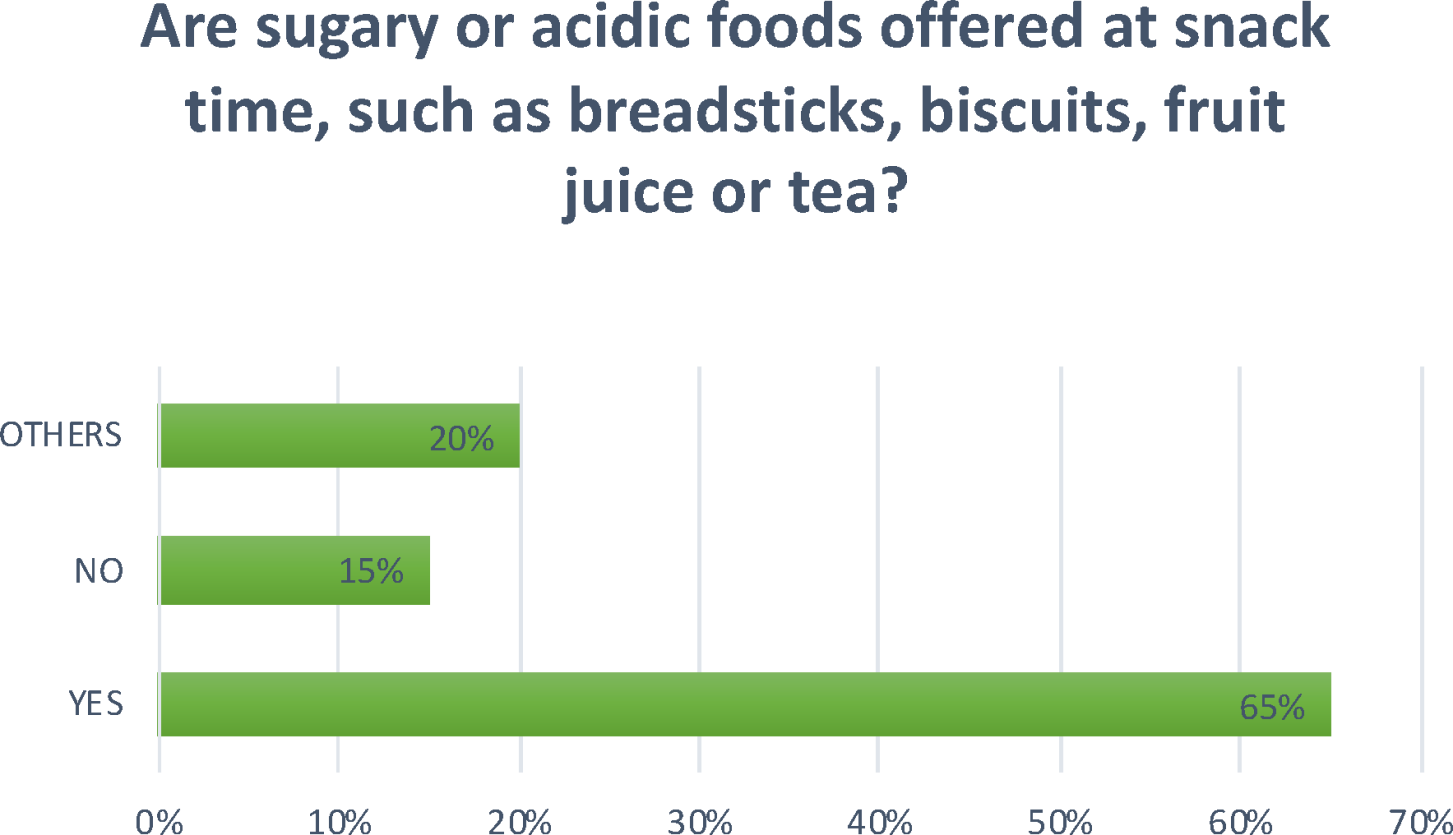
*Are sugary or acidic foods offered at snack time, such as breadsticks, biscuits, fruit juice or tea?* The answers were varied: 65% answered yes and 15% answered no. The remaining 20% is divided between those that offer biscuits and crackers once a week, that offer them only as an afternoon snack, and that serve cake made by the canteen cook once a week.

**Question 11 Figure 11:**
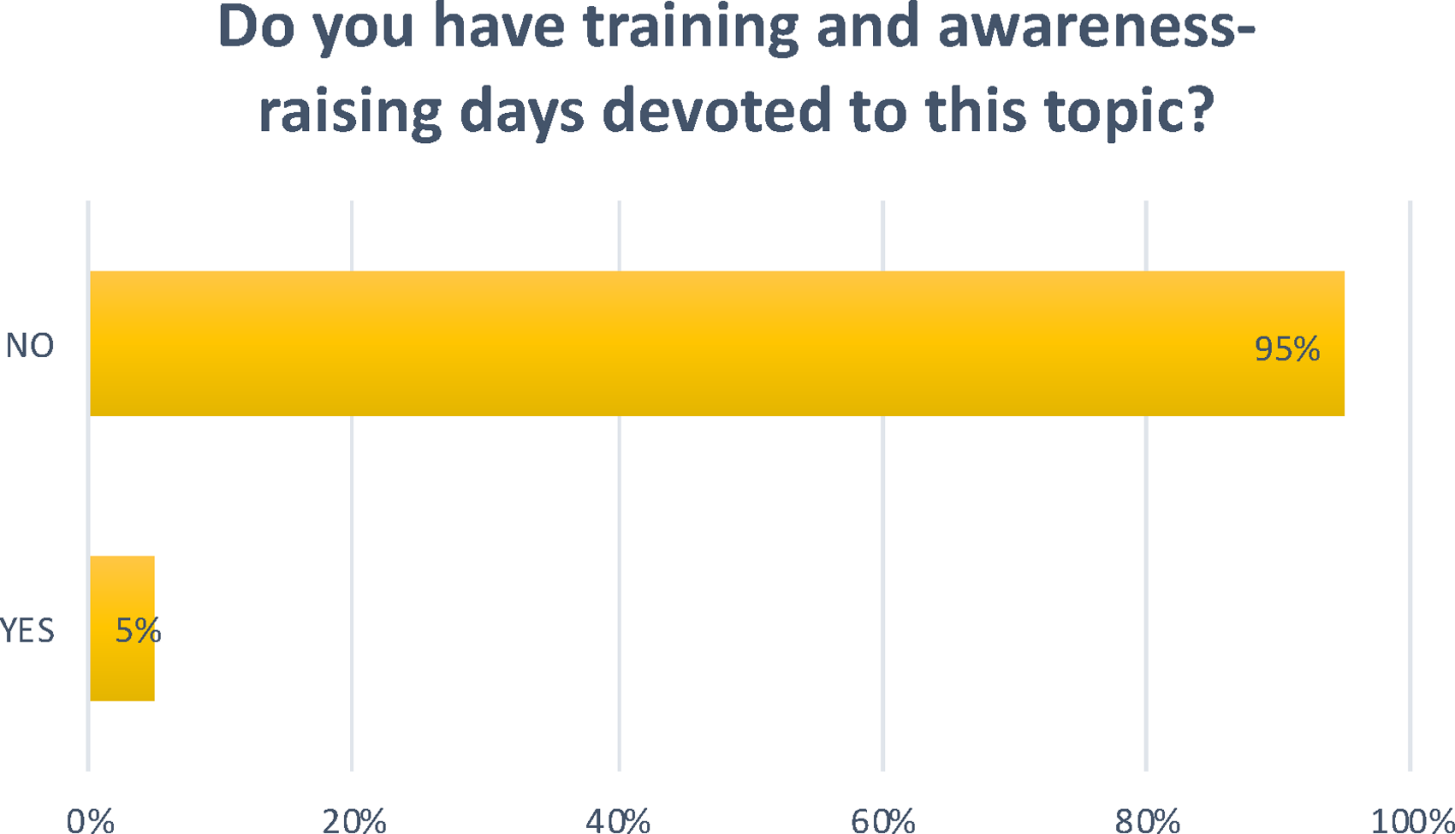
*Do you have training and awareness-raising days devoted to this topic?* 95% replied no; 5% replied yes.

**Question 12 Figure 12:**
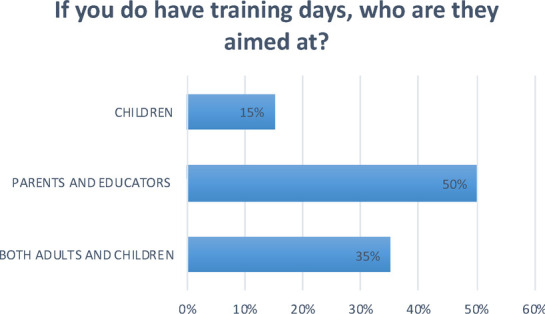
*If you do have training days, who are they aimed at?* 50% of nurseries aim training at adults, such as parents and educators; 15% only target the children. 35% target both adults and children.

**Question 13 Figure 13:**
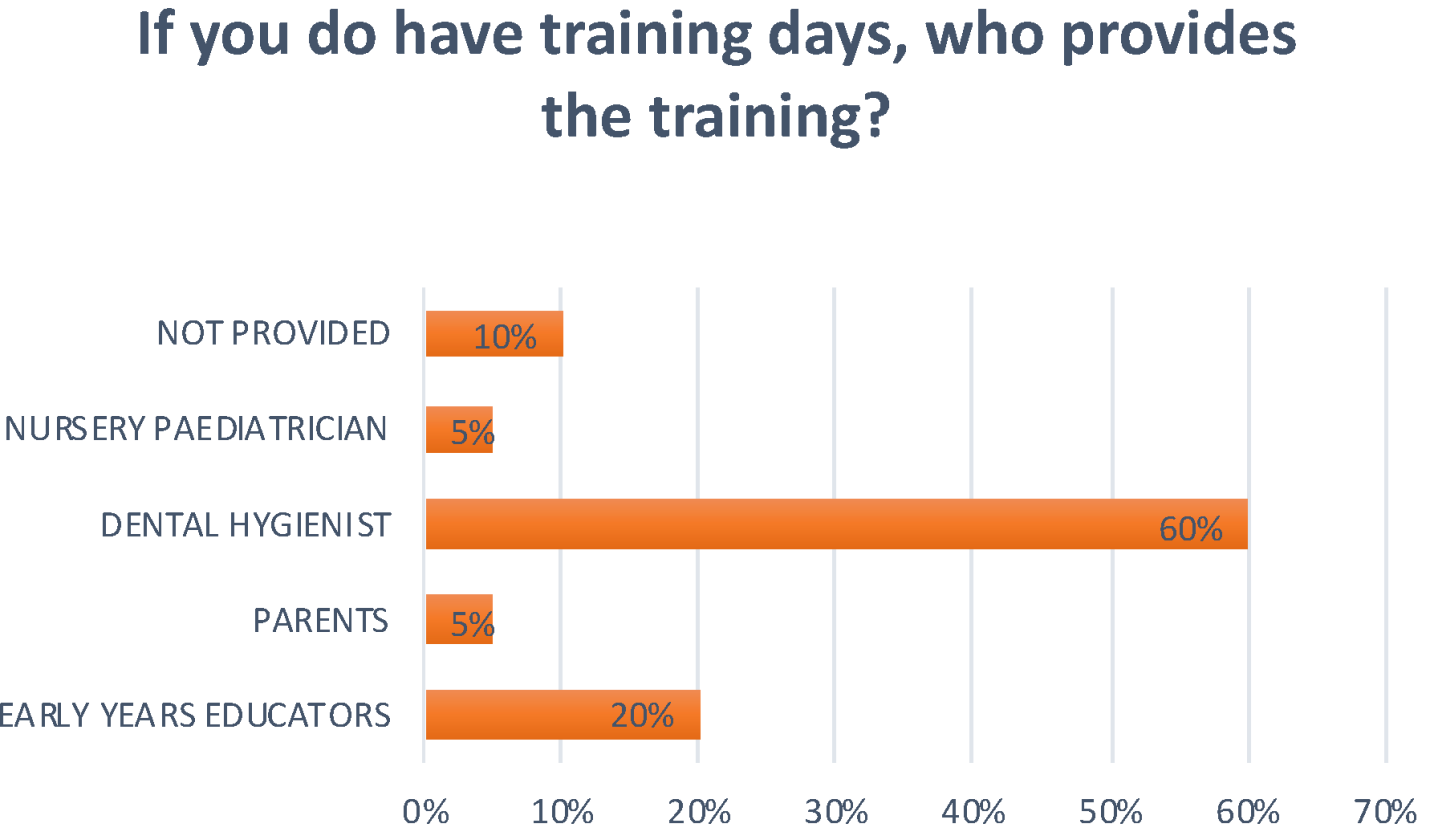
*If you do have training days, who provides the training?* 20% replied early years educators; 5% replied parents; 60% replied a dental hygienist; 5% replied the nursery paediatrician; 10% replied that training was not provided;

**Question 14 Figure 14:**
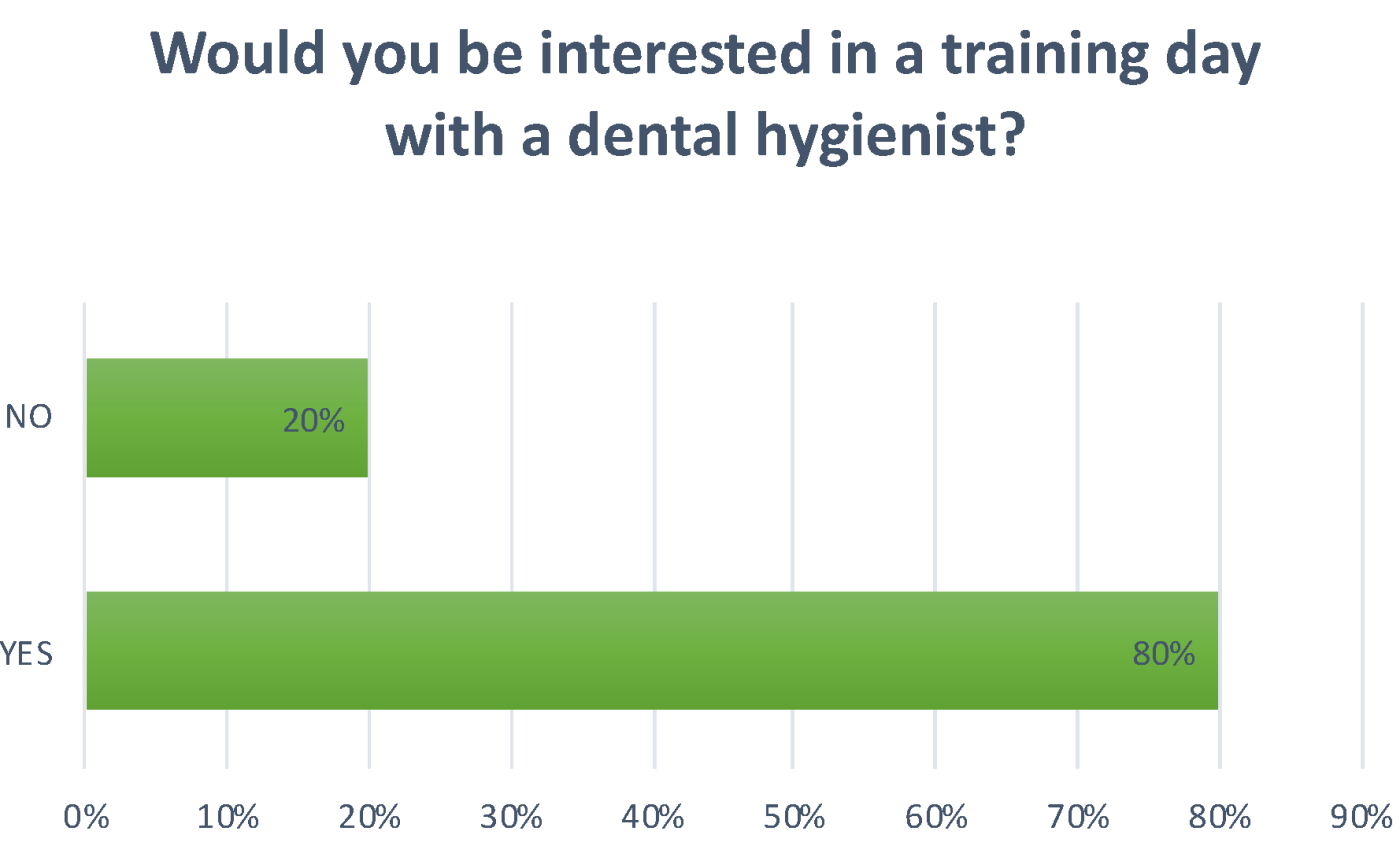
*Would you be interested in a training day with a dental hygienist?* 80% replied yes; 20% replied no.

## Discussion

The age group ranging from 0 to 3 years is complex to analyze, and there are very few articles on oral hygiene in children under the age of three, as confirmed by a study in the United States highlighting the difficulty of finding studies that allow comparison of results in very young children (1).

The aim of this work was to assess the level of attention to oral prevention in nurseries in the city and province of Pavia, the care routines in place, and the knowledge of this topic among educators. The answers obtained through the questionnaire show that there is a lack of clear knowledge about oral hygiene in the age group 0–3 years: 45% of educators believe that oral hygiene procedures should be started only after the eruption of the deciduous teeth, a result which shows that there is still a lot of misinformation about oral care in very young children.

It is important to be aware that tooth decay can occur in children aged 0–3 years. An Italian Ministry of Health handbook, published in 2011, reports statistics showing that 21.6% of children between 0 and 3 years have at least one decayed deciduous tooth (2);. In the United States, carious lesions of childhood (lesions affecting infants aged up to 71 months) are reported in 23% of children, and in China the rate rises to 60% (3).

If good oral hygiene habits are established right from the start, both by parents and by educators, this can facilitate their subsequent acquisition by the child, and their maintenance in adult life.

In most of the nursery schools surveyed using the questionnaire, recreational activities, involving the use of illustrated books, games, puppets, worksheets and drawings, are used to encourage oral hygiene. This is very positive as this approach can certainly help children to see tooth and oral cavity cleaning as a game, and therefore accept it more readily.

Studies have shown that children's oral health can be greatly influenced by that of their parents/ caregivers and the frequency with which the latter have dental checks. A questionnaire-based study evaluating parents' oral health and oral hygiene habits at home reported that children are affected by their parents' knowledge, particularly that of the mother (4). In another study that took into account the frequency of dental checks, parents or caregivers who had checks at least once a year also had their child seen by a dentist in the first three years of life. Conversely, those who did not undergo dental examinations for at least a year did not have their child attend even one dental examination in the first three years of life (1).

A United States study published in 2015 assessed oral health in young children (aged 18–60 months) whose teeth were brushed by their parents: more than half of the parents reported having difficulty brushing their children's teeth and always having to “fight” with them. This shows that it is adults, in particular, that need to be motivated; it is therefore always useful to suggest strategies that might help adults brush their children's teeth more easily, overcoming any resistance (5).

A recent study in France highlighted the importance of preventive measures and oral health education programs for children and their family (6). Respondents were also asked to indicate whether adding this kind of activity might help parents manage children's oral health, and 95% felt that it would; in fact, the more a child gets used to performing certain activities, the more confidently they will repeat them. This is why it is important to establish what parents and educators know about this topic.

At the facilities that implement oral hygiene practices, this activity is mainly carried out after lunch, and only at some nurseries after each meal; most use toothbrushes and toothpaste, while gauze is not very commonly used, which seems to suggest that, as previously mentioned, there is still a considerable amount of misinformation.

Although 80% of the kindergartens surveyed consider it important to add oral hygiene to the activities carried out at their facilities, this practice does not seem to be easily incorporated into the daily routine, probably for different reasons such as a lack of knowledge of the subject or difficulty managing the children.

There emerged considerable differences between the nurseries in the use of toothpaste: in some of them, toothpaste use is not managed in any particular way and children are given complete autonomy; in others fluoride toothpaste is avoided, while others avoid the use of toothpaste altogether; at some nurseries they try to teach children to spit it out.

Another key aspect in prevention is diet. Frequent brushing has a positive effect, reducing the risk of the onset of carious lesions, but only a correct diet associated with good oral hygiene can significantly reduce cases of carious lesions in early childhood (7). In 65% of the nurseries surveyed, foods containing simple sugars are given. Courses on oral hygiene were found to be neither widespread nor carried out by a professional, such as a dental hygienist. This should instead be encouraged.

## Conclusions

The replies collected in this research indicate that information on oral hygiene and on how to maintain it in infants and children aged 0–3 years are not yet widespread in area surveyed.

In view of this, it appears necessary to develop preventive programs geared at improving the level of knowledge and awareness of children's oral health among the adults who care for them. In the area surveyed, there is a need to encourage meetings with parents and educators, where it can be explained in simple terms how and why carious lesions form and how to prevent them, stressing the importance of home oral hygiene and also providing dietary education to further raise people's awareness of this extremely important topic.

## Data Availability

The original contributions presented in the study are included in the article/Supplementary Material, further inquiries can be directed to the corresponding author/s.

## Questionnaire

Hello. I am a student of dental hygiene at the University of Pavia. In the past I have worked as an early years educator in the province of Pavia and this led me to become interested in learning about oral hygiene in nurseries. I am sending you this questionnaire because I would like to find out about oral hygiene practices within these educational facilities.

My aim is to promote dental prevention, as many studies show that tooth decay is common in preschool children. This is a simple questionnaire that should take no more than 5 min to complete, but it will be useful to me for generating statistics and, in the future, for creating courses designed to help prevent tooth decay at this very young age.

Please give honest answers: the questionnaire is anonymous.

I hope you will find this project interesting and above all useful. Thank you.

Name of the nursery (optional)

How many children are there on average at the nursery? * Your reply

Is oral hygiene practised at your nursery? *

- Yes
- No
- Other

At your nursery, do you do any activities encouraging oral hygiene? *

- Puppets
- Games
- Books
- No
- Other

At what age do you think oral hygiene routines should be started? *

- At kindergarten
- Before the milk teeth come through
- After the milk teeth come through

Do you think it would be useful to add this activity at the nursery? *

- Yes
- No

Do you think adding this activity at the nursery could be helpful to parents? *

- Yes
- No

If a programme is in place, what is used? *

- Toothbrushes
- Toothpaste
- Gauze

If toothpaste is used, how do you manage rinsing? *

- A non-fluoride toothpaste is used
- No toothpaste is used
- Trying to teach children to spit
- There is no management of rinsing

If oral hygiene routines are followed, when in the day? *

- No oral hygiene routines are followed
- After every meal
- After lunch

Can the food provided be defined “carioprotective”, i.e., low in simple sugars? *

- Yes
- No

Are sugary or acidic foods offered at snack time, such as breadsticks, biscuits, fruit juice or tea? *

- Yes
- No
- Other

Do you have training and awareness-raising days devoted to this topic? *

- Yes
- No

If you do have training days, who are they aimed at? *

- Only adults, such as parents and educators
- Children and parents
- Children

If you do have training days, who provides the training?

- Dental hygienist
- Educators
- Parents
- Other

Would you be interested in a training day with a dental hygienist? Your reply
